# High-throughput multiplexed autoantibody detection to screen type 1 diabetes and multiple autoimmune diseases simultaneously

**DOI:** 10.1016/j.ebiom.2019.08.036

**Published:** 2019-08-22

**Authors:** Yong Gu, Zhiyuan Zhao, Kathleen Waugh, Dongmei Miao, Xiaofan Jia, Jeremy Cheng, Aaron Michels, Marian Rewers, Tao Yang, Liping Yu

**Affiliations:** a Barbara Davis Center for Diabetes, University of Colorado School of Medicine, Aurora, CO, United States of America; b Department of Endocrinology, First Affiliated Hospital of Nanjing Medical University, China

**Keywords:** Autoantibodies, Multiplex assay, Diabetes, Autoimmune diseases, Biomarker

## Abstract

**Background:**

Islet autoantibodies (IAbs) are the most reliable biomarkers to assess risk of progression to clinical type 1 diabetes (T1D). There are four major biochemically defined IAbs currently used in clinical trials that are equally important for disease prediction. The current screening methods use a radio-binding assay (RBA) for single IAb measurement, which are laborious and inefficient for large-scale screening. More importantly, up to 40% of patients with T1D have other autoimmune conditions that can be identified through relevant autoantibody testing. Thus, there is a need to screen for T1D and other autoimmune diseases simultaneously.

**Methods:**

Based on our well-established electrochemiluminescence (ECL) assay platform, we developed a multiplexed ECL assay that combines 7 individual autoantibody assays together in one single well to simultaneously screen T1D, and three other autoimmune diseases including celiac disease, autoimmune thyroid disease and autoimmune poly-glandular syndrome-1 (APS-1). The 7-Plex ECL assay was extensively validated against single antibody measurements including a standard RBA and single ECL assay.

**Findings:**

The 7-Plex ECL assay was well correlated to each single ECL autoantibody assay and each RBA.

**Interpretation:**

The multiplexed ECL assay provides high sensitivity and disease specificity, along with high throughput and a low cost for large-scale screenings of T1D and other relevant autoimmune diseases in the general population.

**Fund:**

JDRF grants 2-SRA-2015-51-Q-R, 2-SRA-2018-533-S-B, NIH grants DK32083 and DK32493. NSFC grants 81770777.

**Research in context**

**Evidence before this study**

The current screening methods for type 1 diabetes use a radio-binding assay for single islet autoantibody measurement, which are laborious and inefficient for large-scale screening. More importantly, up to 40% of patients with T1D have other autoimmune conditions that can be identified through relevant autoantibody testing. Unfortunately, there is no easy and inexpensive tool to screen for these conditions.

**Added value of this study**

We have developed and validated a simple multiplexed electrochemiluminescence (ECL) assay that combines 7 autoantibody assays in one single well to screen for type 1 diabetes and multiple relevant autoimmune diseases simultaneously with high throughput and low cost. With this new platform, the multiplexed ECL assay can be built in customized panels with different numbers, up to 10, and combinations of autoantibodies according to the needs of clinical settings.

**Implications of all the available evidence**

The multiplexed ECL assay provides high sensitivity and disease specificity, along with high throughput and a low cost for large-scale screenings of type 1 diabetes and other relevant autoimmune diseases in the general population.

Alt-text: Unlabelled Box

## 1 Introduction

Type 1 diabetes (T1D) is one of the most common chronic childhood diseases. Although T1D mainly results from T-lymphocyte mediated destruction of insulin producing beta cells within pancreatic islets, appearance of islet autoantibodies (IAbs) in the peripheral blood is currently the most reliable marker to detect the autoimmune process leading to clinical T1D [1]. Autoantibodies directed against insulin (IAA), glutamic acid decarboxylase-65 (GADA), insulinoma antigen 2 (IA-2A), and zinc transporter-8 (ZnT8A) are routinely measured. Currently, there are approximately 1·4 million people with T1D in the United States. Strikingly, the incidence of is increasing worldwide at 3–5% each year and has doubled in the last two decades, especially in young children [2,3]. IAbs usually appear years before overt clinical disease and nearly all children with the presence of ≥2 IAbs develop clinical T1D when followed over time [4]. The clinical classification of T1D has recently been re-defined to begin with the presence of ≥2 IAbs [5]. Children at risk for T1D need to be identified prior to symptom onset to 1) prevent life-threatening diabetic ketoacidosis, 2) define individuals at high risk for intervention studies, and 3) identify environmental triggers for the onset of islet autoimmunity.

Up to 40% of T1D patients develop an additional autoimmune disorder, and one in four children at risk for T1D in the Diabetes Autoimmunity Study in the Young (DAISY) develop islet, celiac, thyroid or rheumatoid autoimmunity [[6], [7], [8]]. Unfortunately, there is no easy and inexpensive tool to screen for these conditions. In a large effort, all DAISY and TEDDY (The Environmental Determinant of Diabetes in the Young) study participants are screened for autoantibodies to tissue transglutaminase (TGA) for celiac disease autoimmunity. Persistent TGA positivity and celiac disease are secondary endpoints in both studies [9,10], and the TEDDY study has recently initiated screening for autoimmune thyroid disease including antibodies directed against thyroid peroxidase (TPOA) and thyroglobulin (ThGA) in subset of samples (unpublished data). People with positive TPOA and/or ThGA are at risk for developing autoimmune thyroid disease. Autoantibodies to interferon-α (IFNαA) are very specific for autoimmune poly-glandular syndrome-1 (APS-1). All of these autoimmune diseases, including T1D, often begin in childhood.

Through many efforts [11,12], the radio-binding assay (RBA) has been well established as a current ‘gold’ standard assay for measuring IAbs. However, standard RBAs have several important limitations including sensitivity for early detection, disease specificity for positive predictive values, lack of an ability to multiplex, and the use of radiation, which limits widespread clinical adoption. The enzyme-linked immunosorbent assay (ELISA) has long been the primary tool for detection of analytes of interest in biological samples for both life science research and clinical diagnostics. However, ELISA assays (binding of antigen to plate and detection of bound autoantibody with labeled anti-antibodies) have proven difficult to develop and to date only one ELISA-based assay, ElisaRSR™ for GADA, IA-2A, and ZnT8A, has demonstrated sensitivity and specificity similar to the RBA (www.rsrltd.com).

Recently, we developed a novel electrochemiluminescence (ECL) assay to measure IAbs and other autoimmune disease autoantibodies without the need for radiation [[13], [14], [15]]. Through extensive validation in multiple large clinical trials including TEDDY, DAISY, and TrialNet, the ECL assays for IAbs have demonstrated with both higher sensitivity and specificity [[16], [17], [18], [19], [20]], compared to current RBAs. In multiple Islet Autoantibody Standardization Program (IASP) workshops, the IAb ECL assays have been one of the best performing assays. The ECL assay identified IAbs earlier in young children who were followed from birth to clinical T1D. Importantly, ECL assays can define disease-specific IAbs by detecting higher-affinity autoantibodies than those in RBAs. At present, many large scale national intervention trials to prevent T1D are in progress and a wider screening of IAbs in the general population, especially in young children, is needed. Therefore an assay with high throughput that detects disease specific autoantibodies is a critical need for large scale population screening. Here we report a simple multiplexed ECL assay that is based on our extensively validated platform of a single ECL assays. It combines 7 autoantibody assays in one single well needing only six μl of serum to screen for T1D and multiple relevant autoimmune diseases simultaneously.

## 2 Materials and methods

### 2.1 Subjects

Serum samples were randomly selected for three independent cohorts among newly diagnosed patients with T1D (at least one IAb positive) within two weeks of diagnosis at the Barbara Davis Center for Diabetes. Two cohorts of age and sex matched non-diabetic controls were included from the general population cohort in the DAISY study. All control samples were selected as being negative for all four IAbs in previous tests with RBAs. The age and sex distributions for three cohorts of patient and two cohorts of controls used in present study were listed in the Table 1. Signed written informed consents were obtained from participants, and the study was approved by the Institutional Review Board of the University of Colorado.

**Table 1:** Demographics of all cohorts in the present study.

		T1D			Controls	
		Cohort 1 *N* = 186	Cohort 2 *N* = 168	Cohort 3 *N* = 1022	Cohort 1 *N* = 118	Cohort 2 *N* = 1026
Age	Range	1–53	1–55	1–60	1–53	1–52
	Mean	10·5	14·6	10·8	11·4	10·8
	Median	10·0	11·7	9·7	10·4	10·2
Sex	Female	47%	53%	47%	49%	46%

### 2.2 Labeling of antigen proteins with sulfo-tag and biotin

Labeling was performed for each protein with sulfo-tag (MSD) and biotin (Thermo Scientific) as previously reported [21]. Briefly, proteins were mixed with biotin or a sulfo-tag with the molar ratio of 1:5 for antigens with molecular weight ≤10 kDa, and the molar ratio of 1:20 for the antigens with molecular weight >50 kDa in the dark at room temperature (RT) for 1 h. Next the labeled proteins were purified by passing the reaction mixture through a size-exclusion spin column and the labeled antigen concentration (μg/μL) determined.

### 2.3 Checker board assay

This assay is performed to determine optimal concentrations of labeled antigens. In a 96-well PCR plate, half of the plate (6 columns) contains a high antibody positive serum sample and the other half (6 columns) a negative serum sample. Mix 4 μL of serum with 16 μL of PBS in each well and add 10 μL of biotin and 10 μL of sulfo-tag labeled antigen per well. For the concentration of biotin and sulfo-tag labeled antigen, conduct serial dilutions with a vertical 6-column serial dilution for biotin labeled antigen and a horizontal 7-row serial dilution for the sulfo-tag labeled antigen (8th row with 0 concentration). The starting concentrations of a serial dilution for both biotin and sulfo-tag labeled antigens are recommended to be around 1000 ng/mL. The rest of the assay steps will follow the regular ECL assay protocol and the plate will be counted at the end of assay. The best concentrations of biotin and sulfo-tag labeled antigens were identified by selecting a point with the highest or near highest ratio of positive signal to negative signal with consideration of the background from the negative sample.

### 2.4 Create the mixed linker-coupled antigen solution

**Fig. 1:**
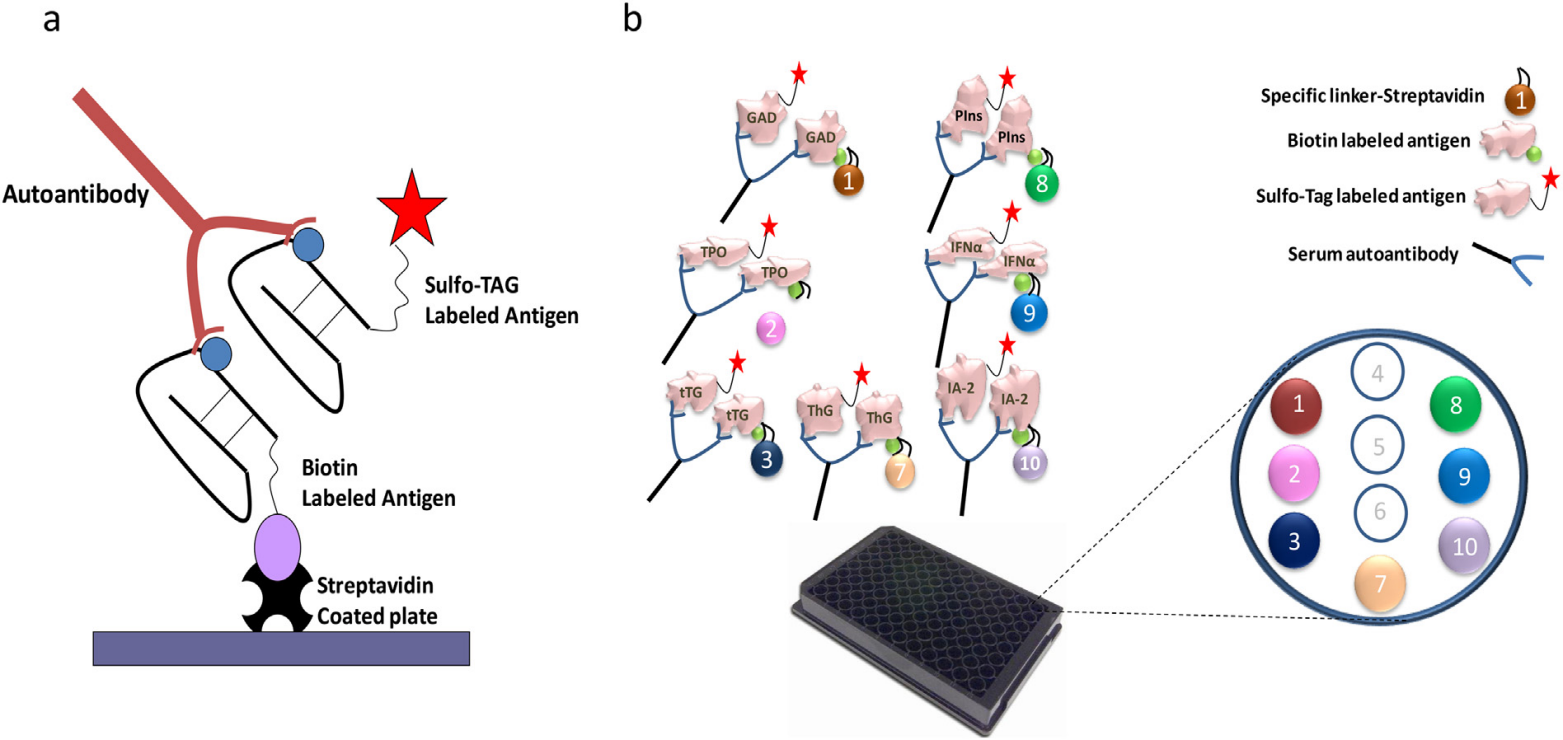
Illustration of the bivalent plate capture ECL assay. Panel a: The autoantibodies in serum will link the sulfo-tagged antigen to the biotinylated antigen which will be captured on the solid phase of the streptavidin coated plate. Detection of plate captured sulfo-tagged antigen is accomplished with electrochemiluminescence. Panel b: The autoantibodies in serum will link the sulfo-tagged antigen to the biotinylated antigen coupled with specific linker which will be captured by the plate. The specifically coupled linker numbers for 7 different antigen proteins are illustrated. Detection of plate captured sulfo-tagged antigens are accomplished with electrochemiluminescence.

To begin, bind 7 different linkers to 7 corresponding biotinylated antigen proteins in separated tubes, respectively. The layout of the 7 active spots on the plate is shown in Fig. 1 (Panel B). Briefly, mix the biotinylated GAD65, TPO, tTG, ThG, proinsulin, INF-α, and IA-2 proteins with streptavidin-conjugated linkers 1, 2, 3, 7, 8, 9, and 10 in separated tubes, respectively, and incubate at RT for 1 h. Next, add stop solution and incubate at RT for 30 min, and then combine all 7 linker-coupled antigens together for use in the multiplex assay.

### 2.5 7-Plex autoantibody ECL assay

Acid treatment of serum is necessary to assay IAA as described in our previous studies [13], and as such we acid treated serum prior to conducting the 7-Plex ECL assay. Briefly, 15 μL of patient serum is mixed with 18 μL of 500 mmol/L of acetic acid. After incubation for 45 min at room temperature, 25 μL of the acid-treated serum solution is transferred to a freshly prepared antigen/neutralization solution consisting of 8·3 μL of 1 M Tris-HCl (pH 9·0) and 35 μL of prepared linker-coupled antigen solution. The mixture is incubated at RT for 2 h on a plate shaker at low speed followed by incubation at 4 °C overnight (>16 h). The same day, a 96-well UPlex plate is blocked with 3% Blocker A (MSD) overnight at 4 °C. The next day, the blocked UPlex plate is washed with PBST (PBS with 0·05% Tween-20) three times followed by the addition of 30 μL of the overnight-incubated mixture onto the plate. After incubation at RT for 1 h, the plate is washed three times with PBST to remove any uncaptured free antigens. Finally, 150 μL/well of 2× Read buffer (MSD) is added and the plate is counted on an ECL reader SQ120 (MSD). Results are expressed as an index (index = [Signal of sample – Signal of Negative Control]/[Signal of Positive Control – Signal of Negative Control]).

### 2.6 Single autoantibody ECL assay

For the ECL-IAA assay, acid treatment of serum samples is required as previously described [13]. All other single autoantibody ECL assays including IA-2A, TGA, TPOA, ThGA, IFNαA are identical to the previously published ECL-GADA assay [14].

### 2.7 Autoantibody assays with RBA or ELISA

GADA and IA-2A RBAs follow the NIH/NIDDK harmonized assay protocol [12]. RBAs for IAA and TGA were developed at Barbara Davis Center for Diabetes and published previously [22,23]. RBAs for TPOA and ThGA were performed with the commercial kits (Kronus). The ELISA assay for IFNαA was conducted following the manufacturer's instructions (Invitrogen).

### 2.8 Statistical analysis

Comparison of the level of the two assays was performed using linear regression, with the single ECL assay or RBA as the independent variable and the multiplex ECL assay as the dependent variable. The assay sensitivity and specificity for each autoantibody in the 7-Plex ECL assay was analyzed by ROC curve analysis including 95% confidence interval (CI) and the area under the curve (AUC) using PRISM 8·0 version software (GraphPad 8·0 Software Inc., San Diego, CA). A two-tailed *p*-value <0·05 is considered significant.

## 3 Results

### 3.1 Optimize and validate each single ECL autoantibody assay on a UPlex plate

The multiplex ECL assay uses a UPlex plate, specifically designed for multiplexed assays and is able to combine up to 10 autoantibody assays in one single well. The multiplex ECL assay is based on a single ECL assay as previously published [[13], [14], [15]]. Fig. 1 illustrates the mechanisms of a single ECL assay (Panel A) and multiplex assay (Panel B). In the present study of the 7-Plex ECL assay, 7 autoantibody assays were combined in one single well including three islet autoantibodies (IAA, GADA, and IA-2A), 2 autoimmune thyroid disease autoantibodies (TPOA and ThGA), celiac disease autoantibodies (TGA), and APS-1 autoantibodies to interferon alpha (IFNaA). Before combining the 7 autoantibody assays into a multiplexed assay, each corresponding autoantibody was tested and optimized in a single autoantibody assay format on a UPlex plate.

#### 3.1.1 Optimization for each single ECL assay on a UPlex plate

A high positive serum sample and a negative control serum sample were utilized to perform optimization of checker board assay for each corresponding autoantibody assay on a UPlex plate. The checker board assay is commonly used for identifying the best assay conditions to obtain the maximum separation of positive from negative signal for which the ratio of two differently labeled antigens and their each optimized concentrations are used in the assay. From the checker board experiments for each of the 7 autoantibody assays, the optimal concentrations of sulfo-tag and biotin labeled antigen were determined and summarized in Supplementary Table 1.

#### 3.1.2 Validation of each single ECL autoantibody assays on a UPlex plate

**Fig. 2:**
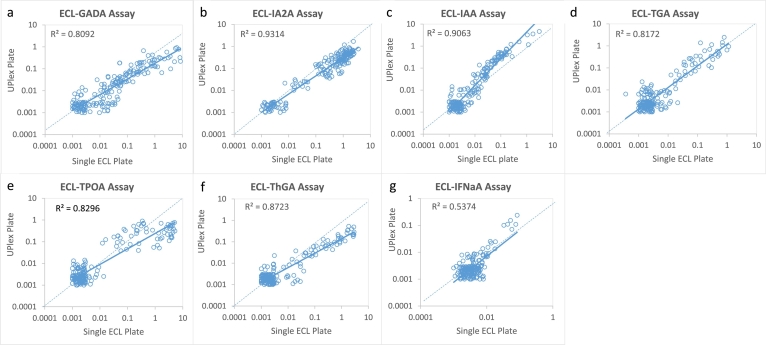
Comparisons in levels of 7 autoantibodies in 186 new onset patients with T1D performed on regular ECL assay plate and UPlex plate. All assays on the UPlex plate were performed with single autoantibody assay. Panels a to g were shown the correlations for GADA, IA-2A, IAA, TGA, TPOA, ThGA, and IFNαA, respectively.

To validate each of the 7 single ECL autoantibody assays on a UPlex plate after optimization, we tested T1D serum samples (*n* = 186) for comparison between the assays. The results of comparisons in levels between single ECL assays performed on regular ECL plate and a UPlex plate for each of 7 autoantibodies are illustrated in Fig. 2. Panels A to G show the correlation for GADA, IA-2A, IAA, TGA, TPOA, ThGA, and IFNaA between two assays. Compared to our established single ECL assays, the levels of autoantibodies detected on UPlex plates were very well correlated for all 7 autoantibodies, R^2^ = 0·81 for GADA, 0·93 for IA-2A, 0·91 for IAA, 0·82 for TGA, 0·83 for TPOA, 0·87 for ThGA, and 0·54 for IFNaA (*p* < 0·0001, linear regression analysis). The R^2^ value for IFNαA is relatively low since only small portion of samples were positive.

### 3.2 Development of 7-Plex ECL assay

**Fig. 3:**
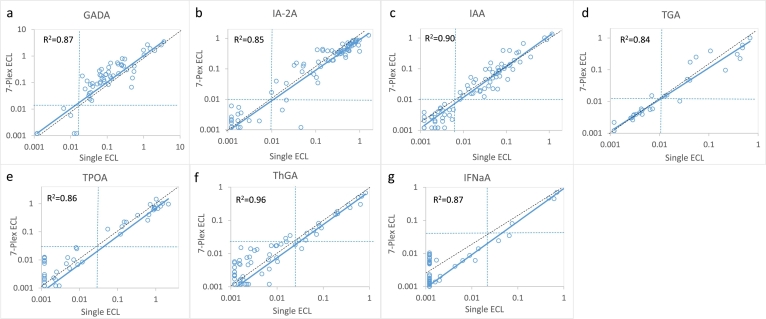
Comparison in levels of 7 autoantibodies in 168 new onset patients with T1D between single ECL assay and the 7-Plex ECL assay. The Panels a, b, c, d, e, f, and g are comparisons for GADA, IA-2A, IAA, TGA, TPOA, ThGA, and TNFαA, respectively. The assay cut-offs were indicated with dotted lines.

**Fig. 4:**
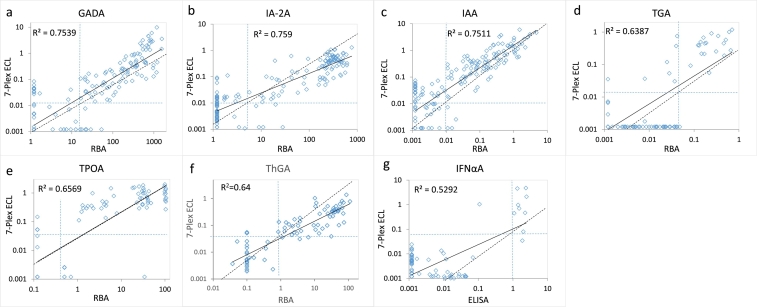
Comparison in levels of 7 autoantibodies in 168 new onset patients with T1D between single RBA assay and the 7-Plex ECL assay. The Panels a, b, c, d, e, f, and g are comparisons for GADA, IA-2A, IAA, TGA, TPOA, ThGA, and TNFαA, respectively. The dotted lines represented the assay cut-offs.

With excellent outcomes in each of the 7 single autoantibody assays on UPlex plates, we then combined these 7 autoantibody assays into one single well to form a 7-Plex ECL assay. We tested 168 new-onset T1D patients and 118 age and sex-matched healthy controls with these assays. The same set of samples were also tested in parallel with our regular single ECL assay and standard RBA or ELISA for each of the 7 autoantibodies. The positive cut-offs for all autoantibodies were set at the 100th percentile from the 118 controls. The results, as shown in Fig. 3, demonstrate that the 7-Plex ECL assay correlated exceptionally well to the levels with each corresponding single ECL assays, R^2^ = 0·87, 0·85, 0·90, 0·84, 0·86, 0·96, and 0·87for GADA, IA-2A, IAA, TGA, TPOA, ThgA, and IFNαA (*p* < 0·0001, linear regression analysis). Similarly, the 7-Plex ECL assay had comparable levels with each corresponding single-antibody RBA or ELISA assay for IFNαA (Fig. 4, R^2^ = 0·75, 0·76, 0·75, 0·64, 0·66, 0·64, and 0·53 for GADA, IA-2A, IAA, TGA, TPOA, ThgA, and IFNα, p < 0·0001 by linear regression analysis). The positivity of the 7-Plex ECL assay and corresponding single ECL assay and RBA or ELISA in 168 T1D patients is summarized in Table 2. The 7-Plex assay retained 100% sensitivity for all autoantibodies and was able to identify more positivity for IAA and TGA samples consistent with our previous findings with ECL assays [13,15]. The few discordant samples for each of 7 autoantibodies were found at low levels, close to the assay cut-offs.

**Table 2:** Number of subjects positive among 168 T1D patients in the 7-plex ECL assay and their corresponding single ECL assay and RBA or ELISA*.

	GADA	IA-2A	IAA	TGA	TPOA	ThGA	IFNaA*
RBA	95	102	101	19	55	51	11
Single ECL	97	110	108	26	58	54	11
7-Plex ECL	97	110	107	26	57	55	11

Asterisk marks the assay for ELISA, while others are RBA.

### 3.3 Validation of 7-Plex ECL assay

To further validate the 7-Plex assay, we tested 1026 new-onset T1D patients and 1022 age and sex-matched healthy control samples (Table 1). Three assays (7-Plex ECL, single ECL, and single RBA or ELISA) were performed in parallel to compare autoantibody levels, sensitivity, and specificity among multiple assay formats. Positive cut off values for each of the 7 autoantibodies for the three different assays were based on the 1022 healthy control subjects tested (Table 3).

**Table 3:** Assay cut-offs based on 1022 general population in the 7-plex ECL assay and their corresponding single ECL assay and RBA or ELISA*.

	GADA	IA-2A	IAA	TGA	TPOA	ThGA	IFNaA*
RBA	20	5	0·010	0·05	0·300	0·300	1·000
Single ECL	0·023	0·010	0·006	0·015	0·030	0·030	0·020
7-Plex ECL	0·015	0·010	0·010	0·015	0·030	0·030	0·080

Asterisk marks the assay for ELISA, while others are RBA.

**Fig. 5:**
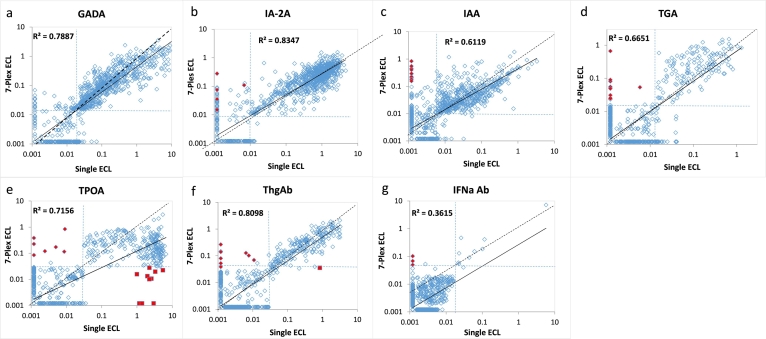
Comparison in levels of 7 autoantibodies in 1026 new onset patients with T1D between single ECL assay and the 7-Plex ECL assay. The Panels a, b, c, d, e, f, and g are comparisons for GADA, IA-2A, IAA, TGA, TPOA, ThGA, and TNFαA, respectively. The dotted lines represented the assay cut-offs. Red close diamonds are those of ‘false’ positives in the 7-Plex ECL assay, but negative in both single ECL and RBA. Red close squares are ‘false’ negatives in the 7-Plex ECL assay caused by ‘Prozon’ phenomenon. (For interpretation of the references to colour in this figure legend, the reader is referred to the web version of this article.)

**Fig. 6:**
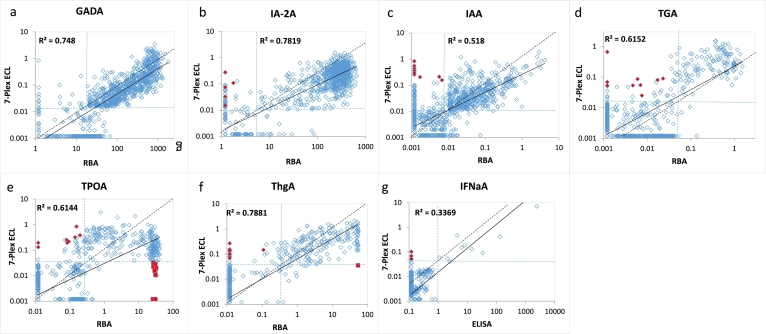
Comparison in levels of 7 autoantibodies in 1026 new onset patients with T1D between RBA (ELISA for INFαA) and the 7-Plex ECL assay. The Panels a, b, c, d, e, f, and g are comparisons for GADA, IA-2A, IAA, TGA, TPOA, ThGA, and INFαA, respectively. The dotted lines represented the assay cut-offs. Red close diamonds are those of ‘false’ positives in the 7-Plex ECL assay, but negative in both single ECL and RBA. Red close squares are ‘false’ negatives in the 7-Plex ECL assay caused by ‘Prozon’ phenomenon. (For interpretation of the references to colour in this figure legend, the reader is referred to the web version of this article.)

The upper limit of normal for all assays was set at the 99th percentile, except for the two thyroid autoantibodies at 95th percentile since thyroid autoantibodies are more commonly present in the general population. Sensitivity and specificity was analyzed by receiver operation curves (ROC) for the 7 autoantibodies in the 7-Plex ECL assay (Supplementary Fig. 1). The area under the curve (AUC) with 95% CI were 0·8649 (0·8479 to 0·8818) for GADA, 0.8924 (0.8769 to 0.9078) for IA-2A, 0.8533 (0.8358 to 0.8708) for IAA, 0.6549 (0.6311 to 0.6787) for TGA, 0.5938 (0.5690 to 0.6186) for TPOA, 0.5778 (0.5531 to 0.6026) for ThGA, and 0.5391 (0.5141 to 0.5641) for IFNαA. The cohort of T1D patients were randomly selected and the positivity of all four non-IAbs were much lower than IAbs as expected, especially for IFNαA, which resulted in overall low sensitivity with low AUC values of ROC curve analysis for four non-IAbs. The sensitivity and specificity of these three different assays are summarized in Table 4. In general, the 7-Plex assay recovered almost all positivity in the T1D patient cohort and had identical specificity in the control cohort. As depicted in Fig. 5, both autoantibody positivity and levels in the 7-Plex assays were well correlated with the single ECL assays for each of the 7 corresponding autoantibodies in the new onset T1D cohort, R^2^ = 0·79 for GADA, IA-2A 0·83, IAA 0·61, 0·67 TGA, 0·72 TPOA, 0·81 ThGA, and 0·36 IFNaA (*p* < .0001, linear regression analysis). The R^2^ value for IFNaA was lower because 99% of the samples were negative. Similarly, the results from 7-Plex assays were well correlated with standard RBA or ELISA for each of the 7 corresponding autoantibodies (Fig. 6, with R^2^ ranging from 0·34 to 0·79, p < .0001 by linear regression analysis). A small number of discordant samples for each of the 7 autoantibodies were found at low levels, close to each assay cut-offs. We noticed that there were 11 control samples (11/1022, 1·1%) positive for multiple autoantibodies that were only seen in the 7-Plex assay but negative in the single assay. The same phenomenon was seen in 9 samples from the T1D patient cohort (9/1026, 0·9%). In addition, 10 samples were found to be high positives for TPOA and one sample with a high positive for ThGA in both the single ECL assay and RBA, but negative in the 7-Plex assay. We diluted these sera and these samples became positive in the 7-Plex assay.

**Table 4:** Assay sensitivity and specificity among 1026 T1D patients and 1022 age and sex matched controls in the 7-plex ECL assay and their corresponding single ECL assay and RBA or ELISA*.

	GADA		IA-2A		IAA		TGA		TPOA		ThGA		IFNαA*	
	Sens	Spec	Sens	Spec	Sens	Spec	Sens	Spec	Sens	Spec	Sens	Spec	Sens	Spec
RBA	73·4%	98·5%	75·2%	99·0%	47·7%	99·1%	12·9%	99·0%	23·9%	95·2%	19·3%	94·7%	1·0%	99·6%
Single ECL	71·6%	99·1%	73·6%	99·3%	48·3%	99·7%	16·0%	98·9%	26·3%	94·9%	20·6%	95·1%	0·8%	99·2%
7-Plex ECL	71·2%	98·9%	77·3%	98·9%	49·5%	98·9%	16·5%	98·7%	26·1%	94·7%	21·1%	94·7%	1·7%	99·1%

Asterisk marks the assay for ELISA, while others are RBA.

## 4 Discussion

Here, we present a high throughput multiplexed autoantibody assay to screen T1D and multiple relevant autoimmune diseases simultaneously. This is the first validated multiplexed autoantibody assay, which is feasible and ready to be used in clinical trials for large-scale population screening. In recent years, many groups have worked to develop a multiplexed assay in a single well to meet the needs of large population screening. A few studies of multiplex autoantibody assays were reported with different technologies in recent years [[24], [25], [26], [27]], but none of these assay platforms has been validated thus far. Very recently, a modified ELISA-based ElisaRSR™ three Screen ICA™ became available. It is a combination assay for measuring three autoantibodies (GADA, IA-2A, and ZnT8A) in one single well [28], but the three Screen is not able to distinguish which of the three autoantibodies are present. The biggest disadvantage of the three Screen ELISA is its inability to include IAA in the assay [29]. IAA has a very high rate of positivity in young children and is considered one of the first IAb in the early stages of islet autoimmunity.

The RBA is the current ‘gold’ standard for all diabetes autoantibody tests worldwide and thus, it is necessary for any newly developed assays to be validated against these standard RBAs for assay sensitivity and specificity. From previous studies, none of the conventional ELISA assays has worked well for any IAb measurements, especially for IAA according to multiple IASP workshops [11]. Interaction of IAbs with their cognate antigen in the fluid-phase is also necessary in a multiplexed assay setting to achieve a proper sensitivity and specificity. In addition, the capacity of specific autoantibody-antigen binding might be a new consideration with our recent experiences in a multiplex assay setting, which has never been an issue in any single antibody assay format. Multiple autoantibody-antigen interactions share space within a single well in a multiplexed format and each labeled antigen must bind enough number of specific antibodies to create a robust signal for detection, which is particularly important when autoantibodies are at low levels.

With the ECL platform, we previously reported a 4-Plex ECL assay using the QuickPlex 4-Spot plate to measure four autoantibodies in one single well for screening T1D and celiac disease [30]. The QuickPlex 4-Spot plate was only able to accommodate a maximum of four autoantibody assays in one single well. With the new UPlex plate system, it is able to create custom multiplex panels of analytes and to multiplex up to 10 different autoantibody assays in one single well with the same amount of serum sample (6 μl) used for a single ECL assay. Recently, we have successfully applied the multiplexed ECL assay in a large trial, Autoimmunity Study in Kids (ASK) [31], designed to screen general population children in the Denver metropolitan area for both T1D and celiac disease using either venous or capillary blood samples. The study aims to screen 50,000 to 60,000 children with over 16,000 children already screened in the last two years. The ASK study is using both standard RBA and multiplex ECL assay (IAA, GADA, IA-2A, and TGA) in parallel. Compared with standard RBA or a single ECL assay, the 4-Plex ECL assay in the ASK study has demonstrated advantages of high throughput, low cost, and low sample volume (only 30% of labor time and lab supply cost, 25% of sample volume) along with excellent assay sensitivity and specificity, identical to our previous reports of single ECL assays [[13], [14], [15], [16], [17], [18], [19], [20],^30^]. The 7-Plex ECL assay developed and validated in the present study needs less sample and labor time resulting in a more cost efficient assay. The data presented in this study illustrates that the 7-Plex assay, compared to either well-established single ECL assay or standard RBA, is able to retain 100% positivity from patients with identical specificity in normal controls. With this new platform, the multiplexed ECL assay can be built in customized panels with different numbers and combinations of autoantibodies according to the needs of clinical settings.

There are some limitations observed in the present study for a multiplexed ECL assay. For the multiplexed assay in a single well, the final dilution of serum in the incubation with labeled antigen cannot be adjusted to the best condition for each autoantibody assays. We observed 11 samples (11/1026) from T1D patients having negative results for particular autoantibodies, 10 negative for TPOA and 1 negative for ThGA, in the 7-Plex ECL assay but showed high positives in both single ECL assay and RBA. All 11 samples became positive in the multiplexed assay if the samples were further diluted in the assay. This result was likely caused by the Prozone phenomenon, which is a false negative response resulting from a high antibody titer interfering with the formation of antigen-antibody conjugation. When a multiplex assay is being developed, a set of samples with both very high and very low titers for each autoantibody are recommended to be pre-tested and serum dilutions optimized to avoid the Prozone phenomenon but without losing the sensitivity. Alternatively, those autoantibody assays with similar optimized conditions, e.g. best condition of serum dilution, should be considered in priority to form a combined assay from which the best assay sensitivity and specificity will be achieved for each of the autoantibodies combined. In the present study, we also observed 11 samples (11/1022) from healthy controls and 9 samples (9/1026) from T1D patients having multiple autoantibodies positive in the 7-Plex assay but clearly negative in the corresponding single ECL assays and RBAs. The reason for the small subset of samples being false positive remains unknown. However, to account for these false positives, all positive results should be repeated with the corresponding single ECL assay as routine laboratory quality assurance/control to confirm positivity, thereby removing these false positives from the multiplexed ECL assay.

In conclusion, we developed a simple high throughput multiplexed ECL assay to simultaneously screen for T1D and multiple relevant autoimmune disease associated autoantibodies. This multiplexed autoantibody assay has been extensively validated, and provides a great tool for general population screening with a high efficiency, low cost, and small sample volume. We anticipate these multiplexed assays will become available in clinics and be easily applied to large population screening in the near future.

The following are the supplementary data related to this article.

Supplementary Fig. 1 ROC analyses for seven autoantibodies, respectively, in 7-Plex ECL assay. Supplementary Fig. 1

Supplementary Table 1 The concentrations of biotin and sulfo-tag labeled antigen proteins in 7-Plex ECL assay. Supplementary Table 1

## Acknowledgements and funding sources

This study is supported by JDRF grants 2-SRA-2015-51-Q-R, 2-SRA-2018-533-S-B, NIH grants DK32083 and DK32493. NSFC grants 81770777. L.Y. has full access to all the data in the study and had final responsibility for the decision to submit for publication.

## Author contributions

Y.G. researched data and wrote manuscript. Z.Z, K.W, D.M, X.J, J.C researched data. A.M and M.R. reviewed/edited manuscript. T. Y and L.Y. designed study, researched data and wrote manuscript.

## Declaration of Competing Interest

None of the authors has any potential financial conflict of interest related to this manuscript.
