# Supplementary figures and images for: High-throughput multiplexed autoantibody detection to screen type 1 diabetes and multiple autoimmune diseases simultaneously
Source: eBioMedicine. 2019 Aug 22;47:365–72. doi: 10.1016/j.ebiom.2019.08.036 (PMC6796526; doi:10.1016/j.ebiom.2019.08.036)

## Slide 1
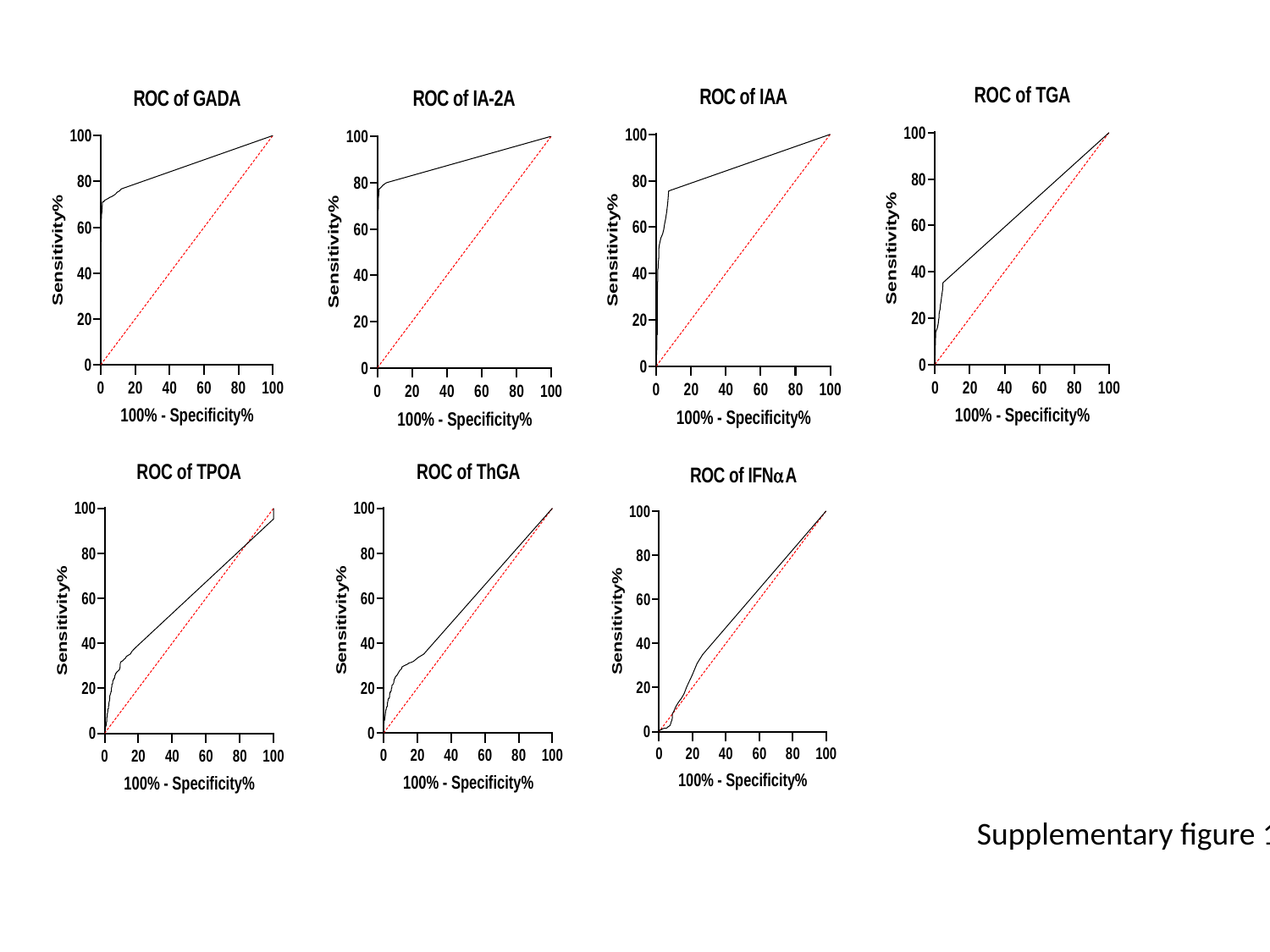

Supplementary figure 1

Supplement: Supplementary Fig. 1 — ROC analyses for seven autoantibodies, respectively, in 7-Plex ECL assay. [file mmc1.pptx]
