# Supplementary material for: High-throughput multiplexed autoantibody detection to screen type 1 diabetes and multiple autoimmune diseases simultaneously
Source: eBioMedicine. 2019 Aug 22;47:365–72. doi: 10.1016/j.ebiom.2019.08.036 (PMC6796526; doi:10.1016/j.ebiom.2019.08.036)
Supplement: Supplementary Table 1 — The concentrations of biotin and sulfo-tag labeled antigen proteins in 7-Plex ECL assay. [file mmc2.docx]

**Supplementary table 1: The concentrations of biotin and sulfo-tag labeled antigen proteins in 7-Plex ECL assay**

|  | **GAD65** | **Proinsulin** | **ThG** | **IFNα** | **TG** | **IA-2** | **TPO** |
| --- | --- | --- | --- | --- | --- | --- | --- |
| Biotin (ng/ml) | 16 | 250 | 31 | 400 | 80 | 40 | 16 |
| Sulfo-TAG (ng/ml) | 8 | 125 | 31 | 100 | 80 | 10 | 8 |
